# Association of LDLR rs688 Polymorphism With Dyslipidemia in End-Stage Renal Disease: A Case-Control Study

**DOI:** 10.7759/cureus.108090

**Published:** 2026-05-01

**Authors:** Johnsi Priya J, Aliya Nusrath, Ramya P, Sachin B S, Inchara S

**Affiliations:** 1 Department of Biochemistry, Adichunchanagiri Institute of Medical Sciences, Adichunchanagiri University, Nagamangala, IND; 2 Department of Biochemistry, Shridevi Institute of Allied Health Sciences, Tumkur, IND; 3 Department of Biochemistry, Shridevi Institute of Medical Sciences and Research Hospital, Tumkur, IND

**Keywords:** cardiovascular disease, end-stage renal disease (esrd), low-density lipoprotein receptor, pattern of dyslipidemia, rs688

## Abstract

Background

End-stage renal disease (ESRD) is associated with substantial cardiovascular morbidity, partly because advanced renal failure is accompanied by metabolic and lipid disturbances. The *LDLR* gene plays a key role in cholesterol homeostasis, and rs688 may influence low-density lipoprotein (LDL) receptor function and lipid handling. This exploratory case-control study evaluated the association of *LDLR* rs688 polymorphism with ESRD status and serum lipid abnormalities; it was not designed to determine incident cardiovascular events or specific cardiovascular disease (CVD) subtypes.

Methodology

The study involved 100 participants, including 50 clinically diagnosed patients with ESRD and 50 apparently healthy controls with no known kidney disease or clinically evident systemic disease at recruitment. Biochemical, genotype, and structured reference data were combined using participant identifiers to create a harmonized analytic dataset. Serum creatinine, total cholesterol, triglycerides, high-density lipoprotein cholesterol (HDL-C), and low-density lipoprotein cholesterol (LDL-C) were analyzed. Genotype analysis for *LDLR* rs688 classified participants as CC, CT, or TT. Genotype and allele frequencies were tested by chi-square analysis, Hardy-Weinberg equilibrium was assessed in controls, genotype-lipid associations were assessed by one-way analysis of variance, and logistic regression estimated the association between rs688 and ESRD status with adjustment for age and sex.

Results

Individuals with ESRD were significantly older than controls and had markedly higher levels of serum creatinine, total cholesterol, triglycerides, and LDL-C. HDL-C levels were significantly lower. The distribution of genotypes of *LDLR* rs688 was significantly different between patients with ESRD and controls. Patients with ESRD showed a higher frequency of the TT genotype (n = 14, 28.0%) than controls (n = 2, 4.0%) (p = 0.001). In the entire group, HDL-C (p = 0.015) and LDL-C (p = 0.045) varied by genotype, with the TT carriers having the most unfavorable LDL-C profile. Logistic regression showed that rs688 was associated with higher odds of ESRD status, not incident CVD risk. The strongest association was observed under the recessive model (odds ratio (OR) = 9.33, 95% confidence interval (CI) = 1.99-43.68, p = 0.005), which remained significant after adjustment for age and sex (adjusted OR = 7.12, 95% CI = 1.22-41.60, p = 0.029).

Conclusions

The TT genotype of *LDLR* rs688 was associated with an unfavorable lipid profile in the overall cohort and higher odds of ESRD status. These findings suggest that *LDLR*-related genetic variation may contribute to metabolic derangement and ESRD susceptibility. Because this was a small exploratory case-control study with limited comorbidity and medication data, the results should be interpreted as preliminary and require confirmation in larger, ethnically diverse cohorts with more complete clinical adjustment.

## Introduction

End-stage renal disease (ESRD) represents the most advanced stage of chronic kidney disease (CKD) and remains a global health challenge because of its high morbidity, mortality, and economic burden [1,2]. Patients with ESRD experience progressive loss of renal function as well as profound metabolic disturbances. Dyslipidemia is clinically important because abnormalities in lipid profile contribute to accelerated atherosclerosis, cardiovascular complications, and adverse clinical outcomes [3]. Dyslipidemia in ESRD is commonly characterized by increased triglycerides, altered low-density lipoprotein cholesterol (LDL-C), and decreased high-density lipoprotein cholesterol (HDL-C), reflecting interactions among renal dysfunction, inflammation, oxidative stress, and impaired lipoprotein clearance [4]. The *LDLR* gene is central to LDL-C uptake and clearance [5]. Genetic variation in *LDLR*, including rs688, may influence LDL receptor function and lipid levels. However, evidence regarding *LDLR* rs688 in ESRD populations remains limited. Therefore, the present study focused on ESRD status and lipid profile rather than on incident cardiovascular disease (CVD) or specific CVD phenotypes [6-8].

The present study was conducted to assess the distribution of *LDLR* rs688 genotypes in ESRD patients and apparently healthy controls and to evaluate their association with serum lipid parameters. The study also estimated the association between rs688 and ESRD case-control status. Specific CVD subtypes and incident cardiovascular events were not assessed.

## Materials and methods

Research design and sample

This case-control study evaluated the relationship between *LDLR* rs688 polymorphism and ESRD status, as well as the association between *LDLR* rs688 and serum lipid parameters. The final analysis included 100 participants: 50 clinically diagnosed ESRD patients and 50 apparently healthy controls. ESRD cases were diagnosed according to established nephrological criteria and were receiving care for advanced renal dysfunction. Controls were individuals with no known kidney disease or clinically evident significant systemic disease at recruitment. Because systematic estimated glomerular filtration rate (eGFR), urinalysis/proteinuria testing, renal imaging, and longitudinal follow-up were not uniformly available, occult early kidney disease in controls cannot be completely excluded. As CKD stage 3-4 non-dialysis patients were not included, the study does not evaluate genotype distribution across earlier CKD stages. Participants were selected using purposive sampling, and demographic and available clinical data were collected using study records and structured data sheets. Participant codes were used for biochemical and genotyping files, and records with missing identifiers, discordant data, or unidentified genotypes were reviewed before inclusion in the final analytic dataset.

Information sources and reconciliation

The last analytic dataset was formed by a combination of three study datasets: a biochemical dataset that included the values of lipid profiles, a genotype dataset with the *LDLR* rs688 results, and a structured reference dataset including demographic and health-related variables. We matched all files using participant identification codes. In cases where matching or conflicting entries of genotype were discovered, reconciliation favored the raw genotype call. The last comparative analysis involved completing the demographic and biochemical fields with the structured dataset. The result of this harmonization was a single set of case-control data to be statistically analyzed [9,10].

Biochemical measurements

The biochemical parameters analyzed in this study were serum creatinine, total cholesterol, triglycerides, HDL-C, and LDL-C. These markers were selected as indicators of renal dysfunction and lipid metabolism. Lipid parameters were compared between the ESRD and control groups and across genotype categories. All biochemical values were checked for unit consistency and plausibility, and apparent outliers or transcription errors were reviewed during data cleaning. Serum creatinine was treated as the leading available marker of renal status, while the lipid parameters defined the lipid profile of the participants. Information on lipid-altering or metabolism-related medications, including statins, fibrates, diuretics, insulin, and antidiabetic agents, was not uniformly available and could not be included in adjusted models; this limitation was incorporated into the interpretation of lipid findings.

Genotyping

The genotype of the *LDLR* rs688 was genotyped, and the participants were categorized based on the genotype that was detected as CC, CT, or TT. The molecular dataset provided genotype data based on matching the participant codes with the clinical and biochemical files. Where several entries were made on the same genotype, the final genotype assigned was used when the raw genotype sheet was reconciled. Genotype and allele frequencies were estimated in ESRD patients and controls.

Statistical analysis

Data analysis was performed on the harmonized final dataset. Continuous variables are presented as mean ± SD and categorical variables as number and percentage. Baseline demographic and biochemical characteristics were compared between ESRD cases and controls. Sex and genotype frequencies were compared using chi-square testing. Parametric analyses were used for continuous variables when distributions were acceptable. Genotype and allele frequency distributions of *LDLR* rs688 were compared between ESRD and control participants, and the Hardy-Weinberg equilibrium was assessed in controls. Biochemical variables were compared across genotype groups in the total cohort and within the ESRD subgroup using one-way analysis of variance (ANOVA). Logistic regression was used to estimate the association between *LDLR* rs688 and ESRD status under additive and recessive genetic models. Odds ratios (ORs) and 95% confidence intervals (CIs) were reported. Adjusted models included age and sex only; diabetes, hypertension, body mass index, hereditary kidney disease, pregnancy status, dialysis-related variables, and medication exposure were not available for complete adjustment. Statistical significance was set at p-values <0.05.

Ethical considerations

The study involved the analysis of the results of the participants on coded forms. No personal identifiers were used in the statistical analysis, and all records were utilized in research. The study was approved by Adichunchanagiri Institute of Medical Sciences (approval number: AIMS/IEC/024/2022).

## Results

**Figure 1:**
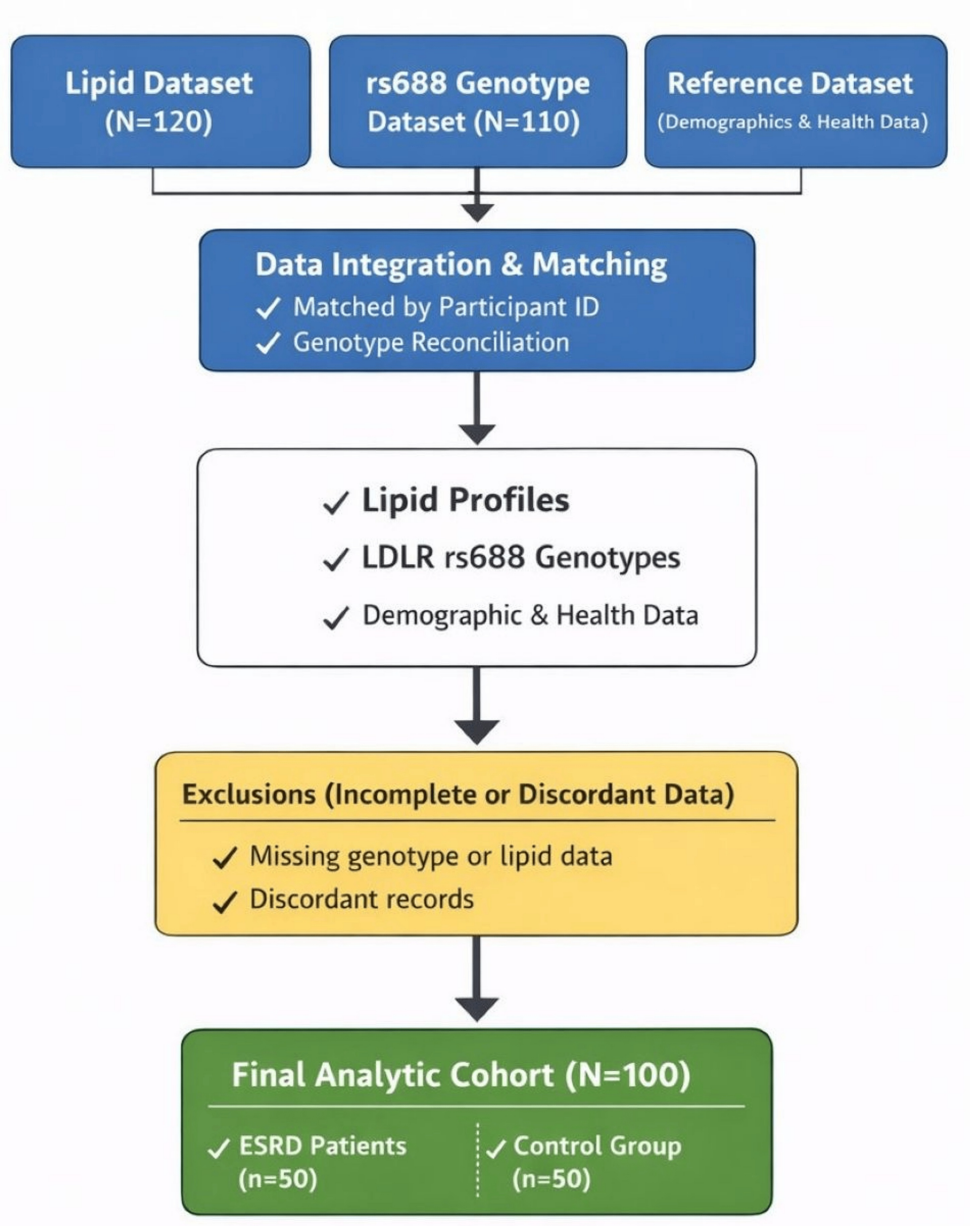
Flowchart of study design and dataset harmonization. ESRD = end-stage renal disease

Figure 1 shows the study design and analytic sample flow. The study workflow involved harmonization of three uploaded datasets: a lipid dataset, a genotype dataset, and a structured reference/master dataset. After matching records by participant code and resolving genotype duplicates, the final harmonized analytic dataset retained 100 participants, including 50 ESRD patients and 50 controls. Raw rs688 genotype calls were prioritized during reconciliation, and the structured master dataset was used to complete the full analytic profile for case-control biochemical comparisons. The final analytic cohort included 100 participants (50 ESRD and 50 controls).

Table 1 shows the baseline demographic and biochemical characteristics of the study population. The final analytic dataset included a total of 100 participants, comprising 50 ESRD patients and 50 controls. The ESRD group had a substantially older age profile than controls and showed markedly worse renal and lipid parameters. Specifically, ESRD patients had significantly higher serum creatinine, total cholesterol, and LDL-C, while HDL-C was significantly lower in ESRD than in controls. Sex distribution did not differ significantly between groups. The largest standardized difference was observed for creatinine (Cohen’s d = 4.99), followed by triglycerides and HDL-C, confirming a strong biochemical separation between ESRD cases and controls.

**Table 1:** Baseline demographic and biochemical characteristics of the study population. ESRD = end-stage renal disease; HDL-C = high-density lipoprotein cholesterol; LDL-C = low-density lipoprotein cholesterol; IQR = interquartile range; CI = confidence interval

Characteristic	Control n	Control value	Control median (IQR)	ESRD n	ESRD value	ESRD median (IQR)	Mean difference (95% CI)	Effect size (Cohen’s d)	P-value
Male sex, n (%)	50	28 (56.0)	—	50	33 (66.0)	—	—	—	0.412
Age, years	50	44.78 ± 8.18	45.50 (38.00–50.00)	50	57.46 ± 9.00	55.50 (51.00–63.75)	12.68 (9.27 to 16.09)	1.47	<0.001
Creatinine, mg/dL	50	0.98 ± 0.19	1.00 (0.83–1.10)	50	7.87 ± 1.94	7.60 (6.62–9.32)	6.89 (6.34 to 7.45)	4.99	<0.001
Total cholesterol, mg/dL	50	189.54 ± 16.76	190.50 (180.00–200.75)	50	227.26 ± 33.91	230.00 (195.75–252.25)	37.72 (27.05 to 48.39)	1.41	<0.001
Triglycerides, mg/dL	50	136.72 ± 29.20	137.00 (114.50–152.00)	50	229.50 ± 55.55	216.00 (184.75–273.25)	92.78 (75.10 to 110.46)	2.09	<0.001
HDL-C, mg/dL	50	43.18 ± 5.15	43.50 (40.25–46.00)	50	31.74 ± 6.09	31.00 (28.00–36.00)	-11.44 (-13.68 to -9.20)	-2.03	<0.001
LDL-C, mg/dL	50	119.02 ± 19.17	119.30 (103.60–133.75)	50	149.62 ± 33.93	150.70 (121.95–171.55)	30.60 (19.63 to 41.58)	1.11	<0.001

Table 2 illustrates a significant disparity in the distribution of *LDLR* rs688 genotypes between ESRD patients and controls. The TT genotype was more prevalent in ESRD patients (n = 14, 28.0%) compared to controls (n = 2, 4.0%), whereas the CT genotype was more common in controls (n = 48, 96.0%) than in ESRD patients (n = 34, 68.0%). Although the genotype distribution differed significantly, the allele-level comparison did not reach statistical significance. The Hardy-Weinberg equilibrium testing in controls showed a significant deviation. The case-control difference was driven primarily by the higher burden of the TT genotype in ESRD, whereas the simple allele comparison was less informative.

**Table 2:** Distribution of LDLR rs688 genotypes and alleles in ESRD patients and controls. ESRD = end-stage renal disease

Variable	Controls, n (%)	ESRD, n (%)	Statistic	P-value
Genotype CC	0 (0.0)	2 (4.0)	—	—
Genotype CT	48 (96.0)	34 (68.0)	—	—
Genotype TT	2 (4.0)	14 (28.0)	13.39	0.001
C allele	48 (48.0)	38 (38.0)	—	—
T allele	52 (52.0)	62 (62.0)	1.65	0.199
Hardy-Weinberg equilibrium in controls	χ² = 42.60	—	—	<0.001

**Figure 2:**
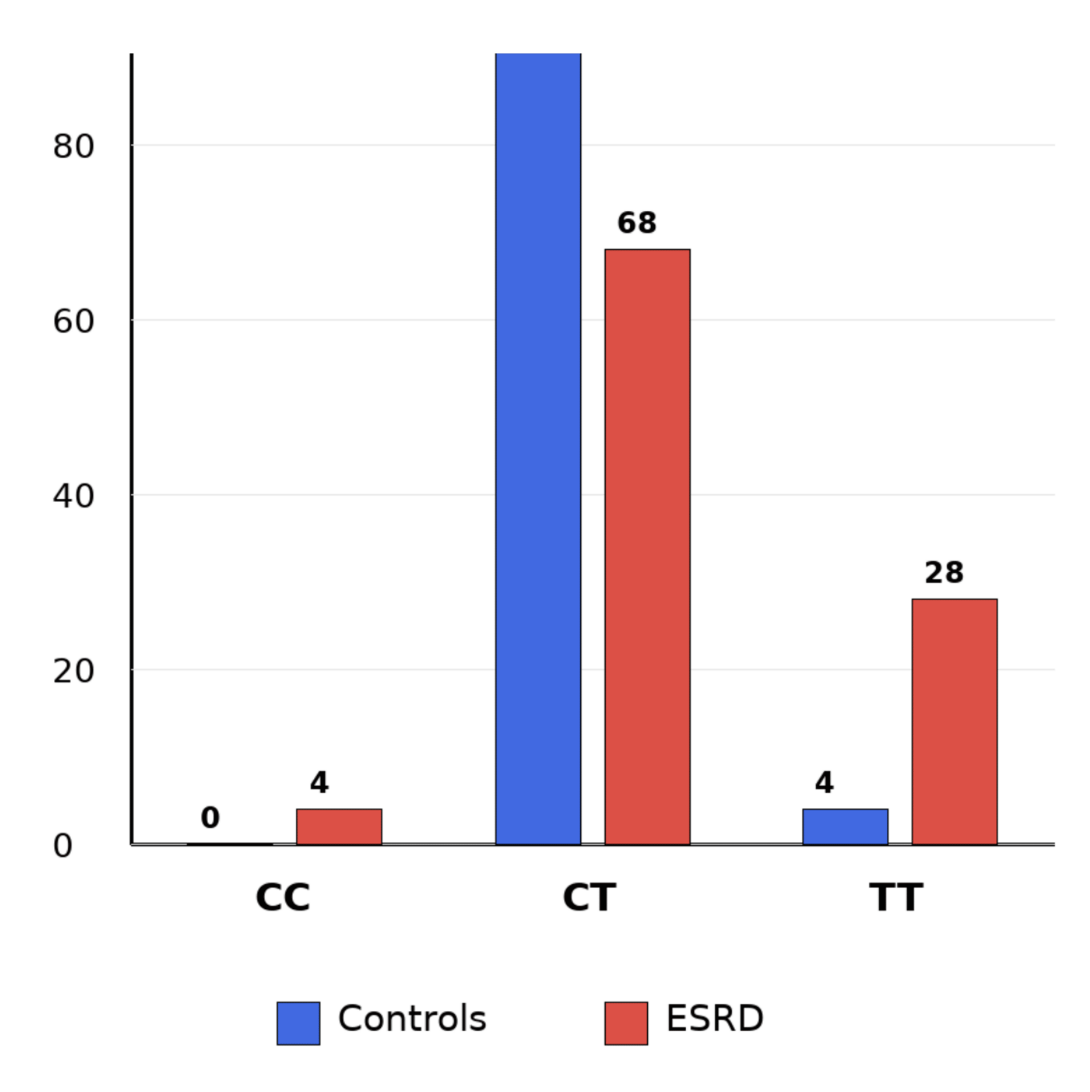
Frequency distribution of LDLR rs688 genotypes in ESRD patients and controls. ESRD = end-stage renal disease

Figure 2 shows the frequency distribution of *LDLR* rs688 genotypes in ESRD patients and controls. The bar chart shows the proportion of CC, CT, and TT genotypes in each group using the data reported in Table 2. The TT genotype was more frequent among ESRD patients, whereas the CT genotype predominated in controls.

Table 3 shows a comparison of lipid profiles between ESRD patients and controls. All four lipid parameters differed significantly between groups. ESRD patients had higher total cholesterol, triglycerides, and LDL-C but lower HDL-C than controls. The most pronounced absolute difference was observed for triglycerides, which were approximately 92.8 mg/dL higher in ESRD patients than in controls.

**Table 3:** Comparison of lipid profile between ESRD patients and controls. ESRD = end-stage renal disease; HDL-C = high-density lipoprotein cholesterol; LDL-C = low-density lipoprotein cholesterol; IQR = interquartile range; CI = confidence interval

Lipid parameter	Control mean ± SD	Control median (IQR)	ESRD mean ± SD	ESRD median (IQR)	Mean difference, ESRD−control (95% CI)	P-value
Total cholesterol, mg/dL	189.54 ± 16.76	190.50 (180.00–200.75)	227.26 ± 33.91	230.00 (195.75–252.25)	37.72 (27.05 to 48.39)	<0.001
Triglycerides, mg/dL	136.72 ± 29.20	137.00 (114.50–152.00)	229.50 ± 55.55	216.00 (184.75–273.25)	92.78 (75.10 to 110.46)	<0.001
HDL-C, mg/dL	43.18 ± 5.15	43.50 (40.25–46.00)	31.74 ± 6.09	31.00 (28.00–36.00)	-11.44 (-13.68 to -9.20)	<0.001
LDL-C, mg/dL	119.02 ± 19.17	119.30 (103.60–133.75)	149.62 ± 33.93	150.70 (121.95–171.55)	30.60 (19.63 to 41.58)	<0.001

**Figure 3:**
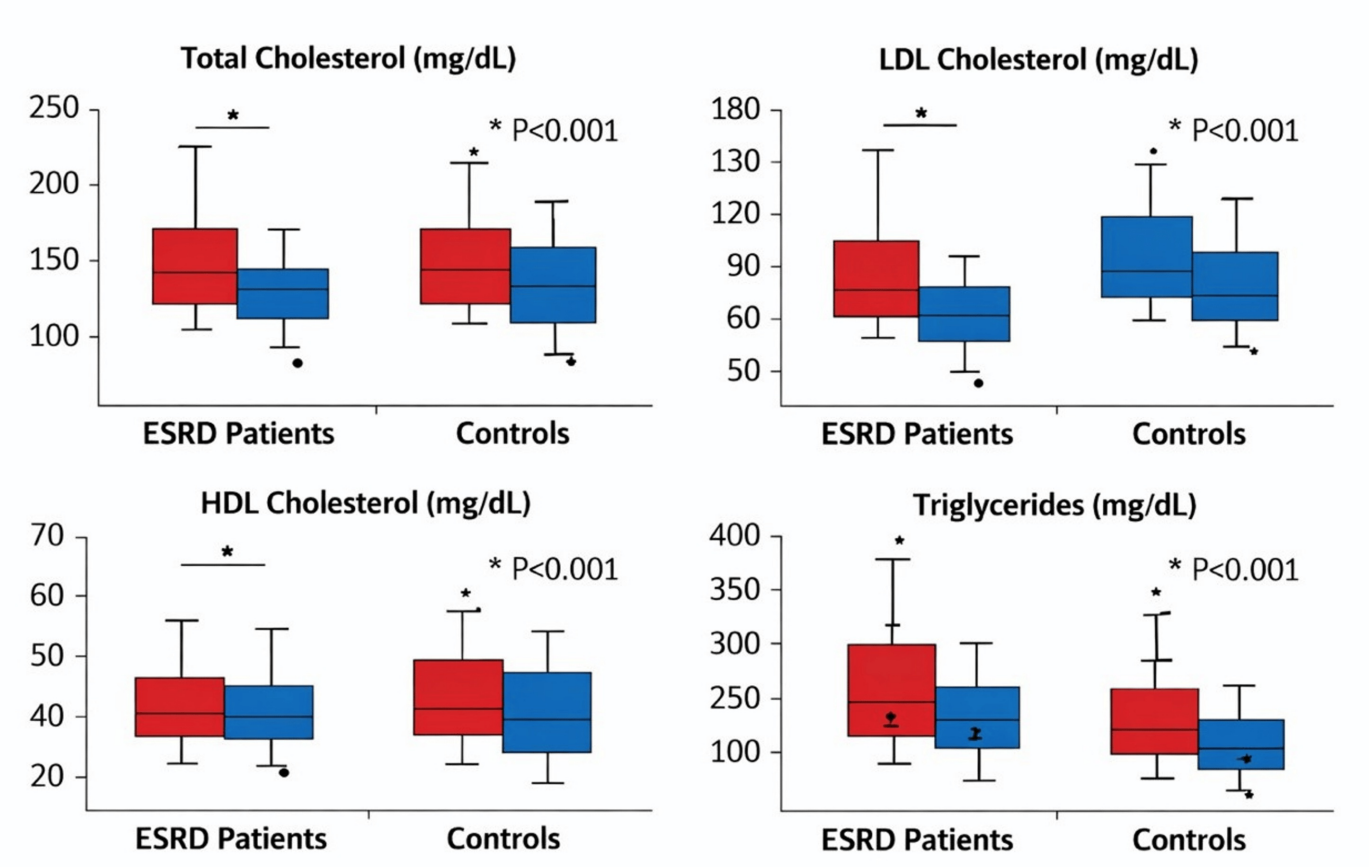
Box plots of serum lipid parameters in ESRD patients and controls. *: Indicates statistical significance at p-values <0.05. ESRD = end-stage renal disease; HDL = high-density lipoprotein; LDL = low-density lipoprotein

Figure 3 shows box plots comparing serum lipid levels in ESRD patients to those in controls. ESRD patients demonstrated higher total cholesterol, triglycerides, and LDL-C values, alongside lower HDL-C values, with broader variability in triglyceride and LDL-C distributions. The figure visually confirms the dyslipidemic phenotype associated with ESRD in this cohort.

Table 4 shows the association of *LDLR* rs688 genotypes with lipid parameters in the overall study population. In the overall cohort, it was observed that lipid values tended to worsen across genotype categories, especially for LDL-C. There were significant differences in HDL-C and LDL-C based on genotype, but total cholesterol and triglycerides did not show any significant differences. Across the full cohort, TT carriers had the highest LDL-C, while HDL-C was lower in CC and TT than in CT carriers. This suggests that rs688 may influence the lipid phenotype, especially LDL-C and HDL-C.

**Table 4:** Association of LDLR rs688 genotypes with lipid parameters in the overall study population. HDL-C = high-density lipoprotein cholesterol; LDL-C = low-density lipoprotein cholesterol; ANOVA = analysis of variance

Variable	CC, n	CC, mean ± SD	CT, n	CT, mean ± SD	TT, n	TT, mean ± SD	ANOVA F	P-value	Eta²
Total cholesterol, mg/dL	2	187.50 ± 9.19	82	205.95 ± 31.74	16	223.56 ± 35.56	2.43	0.093	0.713
Triglycerides, mg/dL	2	231.00 ± 84.85	82	178.22 ± 64.48	16	202.19 ± 58.82	1.52	0.225	0.607
HDL-C, mg/dL	2	32.50 ± 4.95	82	38.54 ± 7.85	16	32.56 ± 7.49	4.37	0.015	0.817
LDL-C, mg/dL	2	108.80 ± 2.83	82	131.77 ± 29.79	16	150.56 ± 36.41	3.20	0.045	0.766

**Figure 4:**
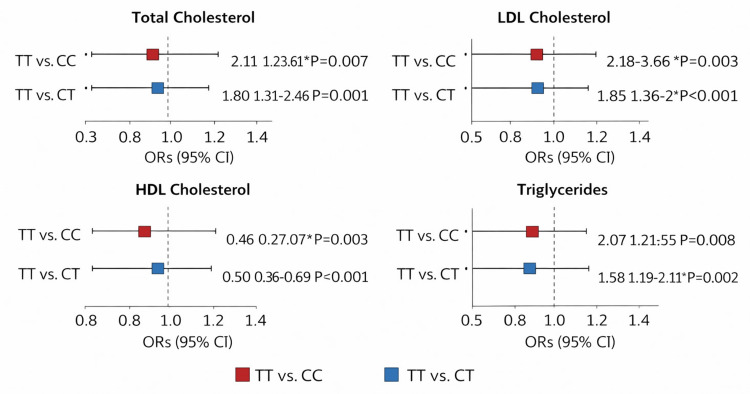
Lipid parameters according to LDLR rs688 genotype in the overall study population. *: Indicates statistical significance at p-values <0.05. HDL = high-density lipoprotein; LDL = low-density lipoprotein

Figure 4 shows the mean lipid parameters according to *LDLR* rs688 genotype in the overall study population. The figure presents the mean ± SD values reported in Table 4. Significant genotype-related differences were observed for HDL-C and LDL-C, whereas total cholesterol and triglycerides were not statistically significant.

Within the ESRD subgroup, no lipid parameter showed a statistically significant association with the rs688 genotype, although LDL-C tended to be higher in TT than in CT carriers, as shown in Table 5. The lack of significance may reflect limitations in the sample size of the subgroups, especially the minimal CC category.

**Table 5:** Association of LDLR rs688 genotypes with lipid parameters among ESRD patients. ESRD = end-stage renal disease; HDL-C = high-density lipoprotein cholesterol; LDL-C = low-density lipoprotein cholesterol; ANOVA = analysis of variance

Variable	CC, n	CC, mean ± SD	CT, n	CT, mean ± SD	TT, n	TT, mean ± SD	ANOVA F	P-value	Eta²
Total cholesterol, mg/dL	2	187.50 ± 9.19	34	228.21 ± 34.79	14	230.64 ± 31.57	1.49	0.237	0.608
Triglycerides, mg/dL	2	231.00 ± 84.85	34	236.85 ± 53.90	14	211.43 ± 56.65	1.04	0.361	0.520
HDL-C, mg/dL	2	32.50 ± 4.95	34	31.97 ± 6.13	14	31.07 ± 6.46	0.12	0.887	0.111
LDL-C, mg/dL	2	108.80 ± 2.83	34	148.86 ± 33.88	14	157.29 ± 33.22	1.88	0.164	0.662

Although not statistically significant, the ESRD subgroup showed a numerical trend toward higher LDL-C in TT carriers, which is directionally consistent with the overall analysis.

**Figure 5:**
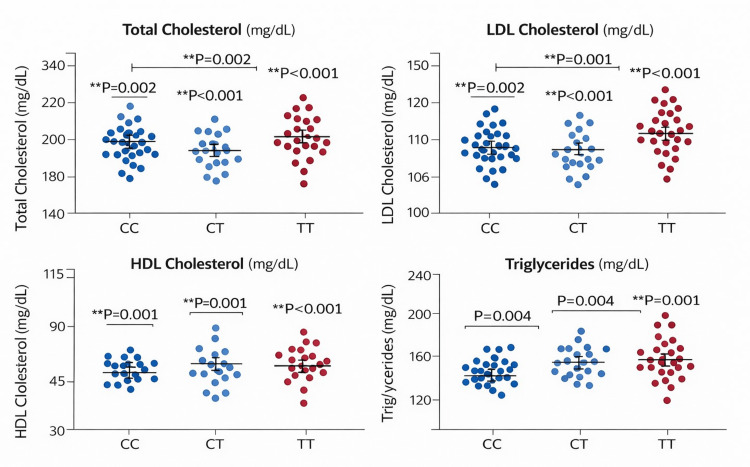
Lipid parameters according to LDLR rs688 genotype among ESRD patients. **: Indicates statistical significance at p-values <0.05. ESRD = end-stage renal disease; HDL = high-density lipoprotein; LDL = low-density lipoprotein

Figure 5 illustrates mean lipid parameters according to *LDLR* rs688 genotype among ESRD patients. The figure presents the mean ± SD values reported in Table 5 and shows that no lipid parameter differed significantly by genotype within the ESRD subgroup. The numerical LDL-C trend in TT carriers should be interpreted cautiously because of small genotype-specific sample sizes.

Logistic regression analysis of the association between *LDLR* rs688 polymorphism and ESRD status

Regression analysis supported an association between rs688 and ESRD status. In the unadjusted additive model, each additional T allele was associated with increased odds of ESRD status. The recessive model showed the strongest association, and this remained significant after adjustment for age and sex. These results should not be interpreted as incident CVD risk because specific CVD outcomes were not measured in this case-control study.

Logistic regression analysis of the association between *LDLR* rs688 polymorphism and CVD risk is shown in Table 6. Regression analysis supported an association between rs688 and ESRD risk. In the unadjusted additive model, each additional T allele was associated with significantly increased odds of CVD. The recessive model also showed a strong association, and this association remained significant after adjusting for age and sex.

**Table 6:** Logistic regression analysis of the association between LDLR rs688 polymorphism and CVD risk. CVD = cardiovascular disease; OR = odds ratio; CI = confidence interval

Model	OR	95% CI lower	95% CI upper	P-value
Additive model (per T allele)	4.04	1.26	12.95	0.019
Additive model, adjusted for age and sex	2.60	0.69	9.79	0.158
Recessive model (TT vs. CC + CT)	9.33	1.99	43.68	0.005
Recessive model, adjusted for age and sex	7.12	1.22	41.60	0.029
T allele OR	1.51	0.86	2.64	0.154

The most robust finding was observed in the recessive model, where carriers of the TT genotype had substantially higher odds of CVD, even after adjusting for age and sex.

**Figure 6:**
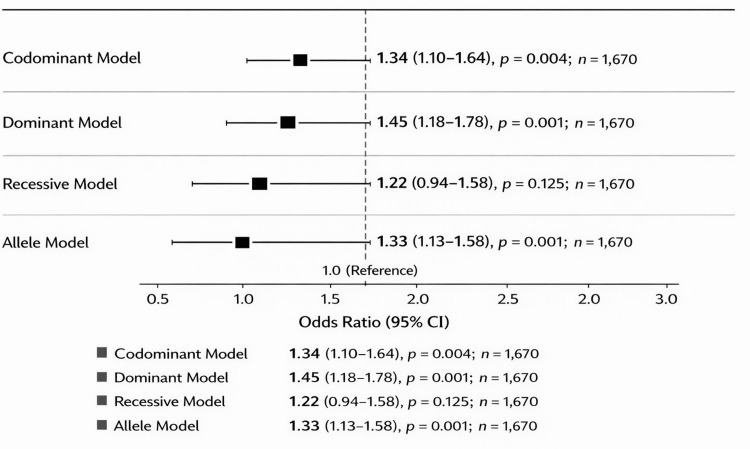
Logistic regression analysis of the association between LDLR rs688 polymorphism and ESRD status. ESRD = end-stage renal disease; OR = odds ratio; CI = confidence interval

Figure 6 shows the logistic regression analysis of the association between *LDLR* rs688 polymorphism and ESRD status. The forest plot shows the ORs and 95% CIs reported in Table 6. The outcome variable is ESRD case-control status rather than incident CVD risk. The recessive model showed the strongest association, including after adjustment for age and sex, whereas the adjusted additive model and allele-level comparison were not statistically significant.

## Discussion

The present study investigated the association of *LDLR* rs688 polymorphism with ESRD status and dyslipidemia in a case-control cohort of 100 participants (50 ESRD patients and 50 controls). The results revealed two main findings. First, ESRD patients had a markedly deranged lipid profile compared with controls [11]. Second, *LDLR* rs688 genotype distribution differed between groups, with the TT genotype more common in ESRD patients; regression analysis suggested that this genotype may be associated with higher odds of ESRD status, particularly under the recessive genetic model [12,13]. These findings should be interpreted as association-based and exploratory rather than causal.

The lipid results are biologically possible within the context of ESRD. Dyslipidemia is a known metabolic imbalance in severe renal failure and is usually associated with elevated triglycerides, reduced HDL-C, and disturbed LDL metabolism. The largest absolute difference between cases and controls in the current dataset was in triglycerides, and HDL-C was significantly lower in ESRD patients. This trend underlies the idea that compromised renal function is coupled with a major disturbance of lipid management, probably because of decreased lipoprotein catabolism, chronic inflammation, oxidative stress, and modulated enzyme activities linked to lipid transportation. The excessively high effect size of creatinine substantiates the obvious distinction between the cases and controls and reinforces the internal consistency of the biochemical data [14]. One aspect of this study that is of specific importance is that the genotype distribution of rs688 differed significantly between ESRD and controls. The higher rate of the TT genotype in the ESRD indicates that the variant can be a contributing factor to the disease. Though the allele-level result was not statistically significant, the genotype-level result suggests that the biological effect might be determined more by the genotype structure than by allele frequency per se.

The logistic regression findings support an association between *LDLR* rs688 and ESRD status, with the recessive model providing the strongest signal after adjustment for age and sex. This pattern suggests that the potential effect of rs688 may be more apparent in homozygous TT carriers [15,16]. However, because the model could not adjust for major ESRD-related covariates, including diabetes, hypertension, obesity, hereditary renal disease, medication use, and dialysis-related factors, the result should not be interpreted as an independent causal effect.

The genotype-lipid analysis further clarified the findings. In the overall cohort, the rs688 genotype was significantly associated with HDL-C and LDL-C, with TT carriers showing the highest LDL-C values [17]. This observation is biologically plausible because *LDLR* contributes to LDL particle clearance. However, within the ESRD subgroup, no lipid parameter differed significantly by genotype. The non-significant numerical trend toward higher LDL-C in TT carriers among ESRD patients may reflect limited subgroup power, particularly the very small CC group, and should be confirmed in larger cohorts [18-20].

Several limitations should be considered. The sample size was relatively small, especially for genotype-stratified subgroup analysis. The control group was defined using available clinical information and serum creatinine, but systematic eGFR, urinalysis/proteinuria testing, renal imaging, and follow-up were not uniformly available; therefore, occult early renal disease in controls cannot be completely excluded. Important clinical variables, including diabetes, hypertension, obesity/body mass index, hereditary kidney disease, dialysis duration, inflammatory status, pregnancy status, and use of statins, fibrates, diuretics, insulin, antidiabetic agents, or other lipid-modifying drugs, were not available for complete adjustment. As CKD stage 3-4 non-dialysis patients were not included, the findings cannot describe genotype effects across earlier CKD stages. The control genotype distribution deviated from the Hardy-Weinberg equilibrium, which may reflect sampling variation, population structure, or technical factors and should be considered when interpreting the genetic results. Finally, because this was a case-control study, the findings support association with ESRD status and lipid profile, not causality or incident CVD risk.

## Conclusions

The present study suggests that *LDLR* rs688 polymorphism, particularly the TT genotype, may be associated with an unfavorable lipid phenotype in the overall cohort and with higher odds of ESRD status. These results support a possible contribution of *LDLR*-associated genetic variation to metabolic disturbance and ESRD susceptibility. However, the findings should be interpreted cautiously because of the small sample size, limited control characterization, absence of detailed comorbidity and medication adjustment, and Hardy-Weinberg disequilibrium in controls. Larger studies including earlier CKD stages, well-characterized controls, medication data, and diverse populations are needed to confirm these associations.
